# Optimization of guidelines for Risk Of Recurrence/Prosigna testing using a machine learning model: a Swedish multicenter study

**DOI:** 10.1016/j.breast.2025.104489

**Published:** 2025-05-07

**Authors:** Una Kjällquist, Nikos Tsiknakis, Balazs Acs, Sara Margolin, Luisa Edman Kessler, Scarlett Levy, Maria Ekholm, Christine Lundgren, Erik Olsson, Henrik Lindman, Antonios Valachis, Johan Hartman, Theodoros Foukakis, Alexios Matikas

**Affiliations:** a Department of Oncology/Pathology, Karolinska Institutet, Stockholm, Sweden; b Theme Cancer, Karolinska University Hospital, Stockholm, Sweden; c Department of Clinical Science and Education, Södersjukhuset, Karolinska Institutet, Stockholm, Sweden; d Department of Oncology, Södersjukhuset, Stockholm, Sweden; e Breast Center, Capio St:Göran's Hospital, Stockholm, Sweden; f Department of Oncology, Ryhov County Hospital, Jönköping, Sweden; g Department of Biomedical and Clinical Sciences, Division of Oncology, Linköping University, Linköping, Sweden; h Department of Immunology, Genetics and Pathology, Uppsala University, Uppsala, Sweden; i Department of Oncology, Faculty of Medicine and Health, Örebro University, Örebro, Sweden

**Keywords:** Adjuvant, Breast cancer, Machine learning, Prosigna, Risk of recurrence

## Abstract

**Purpose:**

Gene expression profiles are used for decision making in the adjuvant setting in hormone receptor-positive, HER2-negative (HR+/HER2-) breast cancer. While algorithms to optimize testing exist for RS/Oncotype Dx, no such efforts have focused on ROR/Prosigna. This study aims to enhance pre-selection of patients for testing using machine learning.

**Methods:**

We included 348 postmenopausal women with resected HR+/HER2-node-negative breast cancer tested with ROR/Prosigna across four Swedish regions. We developed a machine learning model using simple prognostic factors (size, progesterone receptor expression, grade, and Ki67) to predict ROR/Prosigna output and compared the performance regarding over- and undertreatment with commonly employed risk stratification schemes.

**Results:**

Previous classifications resulted in significant undertreatment or large intermediate groups needing gene expression profiling. The machine learning model achieved AUC under ROC of 0.77 in training and 0.83 in validation cohorts for prediction of indication for adjuvant chemotherapy according to ROR/Prosigna. By setting and validating upper and lower cut-offs corresponding to low, intermediate and high-risk disease, we improved risk stratification accuracy and reduced the proportion of patients needing ROR/Prosigna testing compared to current risk stratification.

**Conclusion:**

Machine learning algorithms can enhance patient selection for gene expression profiling, though further external validation is needed.

## Highlights

• Selection of postmenopausal patients with HR+/HER2- N0, breast cancer for gene expression profiling is imprecise.
• Common clinical risk definitions result in under- or overtreatment, or large intermediate risk groups, and excessive testing.
• We show the feasibility of machine learning algorithms to improve patient selection for testing with ROR/Prosigna.
• Further validation in larger external cohorts is needed.

## 1 Introduction

Use of adjuvant chemotherapy following resection of hormone receptor positive, human epidermal growth receptor 2 negative (HR+/HER2-) breast cancer is guided by gene expression profiles (GEP) [1,2]. These tools provide prognostic information independent of clinical or pathologic factors and identify patients at low absolute risk for recurrence, for whom chemotherapy does not improve outcomes [[3], [4], [5], [6]]. Clinical information is also considered [2], either as a trigger to test with specific assays, a component of second generation tools, or for decision making when multigene assays are not available [1,2]. For example, tumor size and grade guide the use of 70-gene/MammaPrint (Agendia Inc., Amsterdam, The Netherlands) [5], whereas Ki67 may aid in the selection of patients for adjuvant chemotherapy when no GEP are available [2,7].

As GEP are widely recommended and used, the cost-benefit aspect becomes central. Different guidelines and risk-stratification algorithms have been suggested to select patients for multigene assay testing to improve cost-effectiveness. The IHC4 score [8], the Anne Arundel Medical Center model [9], the Magee equations [10], and optimized Rochester Modified Magee algorithm [11] have been developed to optimize the use of RS/Oncotype Dx (Exact Sciences, Madison, WI, USA) assay based on histopathologic factors. While ROR/Prosigna (Veracyte, South San Francisco, CA, USA) compares favorably regarding its prognostic capacity [12], its usage, indication, and interpretation lack standardization. Therefore, improved pre-selection of patients needed to test is warranted, mirroring the algorithms designed for RS/Oncotype Dx testing.

We have previously reported on the real-world impact of ROR/Prosigna testing on administration of adjuvant chemotherapy in a Swedish multicenter study [13]. In the present study, we report how patient selection for testing with GEP is affected by clinical risk factors and describe how selection for testing can be optimized by employing machine-learning models that leverage readily available clinicopathologic variables.

## 2 Materials and methods

### 2.1 Study design

**Fig. 1:**
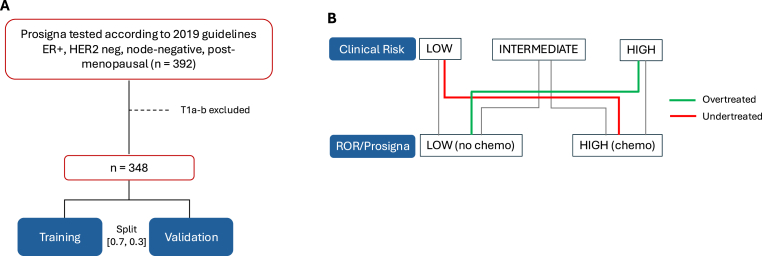
Flowchart of patients included in the study (A). Conceptualization of over- and undertreatment based on ROR/Prosigna risk classification. Clinical risk groups were dichotomized into molecular (ROR) low risk (no indication for chemo) or high risk (indication for chemo). Low clinical risk with high molecular risk (ROR) or high clinical risk with low molecular risk will be undertreated or overtreated respectively, if not tested (B).

All postmenopausal women with HR+/HER2-and node negative breast cancer, with tumor stage T1c or higher, diagnosed between March 2020 and March 2022 and tested with ROR/Prosigna in four Swedish regions (Stockholm, Uppsala, Jönköping, Örebro) were included in the study cohort. The population from these regions constitutes 33 % (3.5 million) of the Swedish population and was therefore considered to form a representative sampling source. All participating centers (Karolinska University Hospital, Södersjukhuset and St:Görans hospital in Stockholm, Akademiska Hospital in Uppsala, Ryhov County Hospital in Jönköping, and University Hospital in Örebro) follow the national guidelines of biomarker evaluation and participate in internal and external quality control for breast cancer biomarker testing [14]. The biomarker assessment has been previously described in detail [13]. The pooled study cohort comprises 348 patients with available ROR/Prosigna as shown in Fig. 1a.

According to regional guidelines for GEP to guide clinical decision making during the study period, ROR/Prosigna was broadly recommended for postmenopausal women with node-negative, HR+/HER2-breast cancer during 2019–2021. In 2022, after the end of the study period, these guidelines were updated with new cut-off values for Ki-67 (low <5 %, intermediate 5–30 %, high >30 %) and recommendation to omit testing in patients with pT1bN0 tumors due to the likely low absolute benefit from chemotherapy [15,16]. Both guidelines are presented in detail in Supplementary Tables 1 and 2

The aim was to relate the clinical risk categories according to the guidelines and the clinical risk stratification schemes described hereunder to the actual outcome of the ROR/Prosigna test. ROR/Prosigna low risk (ROR ≤40, no indication for chemotherapy) or intermediate/high risk (ROR >40, indication for chemotherapy) were considered as ground truth. Patients with low clinical risk but indication for chemotherapy according to ROR/Prosigna, or high clinical risk with no indication for chemotherapy according to ROR/Prosigna were considered to be undertreated or overtreated respectively, had they not been tested, as depicted in Fig. 1b.

### 2.2 Model development

We hypothesized that a trained machine learning model could assist to classify patients with clinical intermediate risk into either of low or high molecular risk (based on Prosigna ROR score) as well as lower the number of patients that would be tested with ROR/Prosigna or treated with chemotherapy if not tested.

In order to mitigate bias due to variable local testing routines, we pooled all patients and split the study cohort based on a 0.7, 0.3 proportion rule, balanced in terms of the ground truth (chemotherapy indication according to ROR/Prosigna). The following variables were used to build the model: progesterone receptor expression, tumor size, grade and Ki67. Size and Ki67 exhibited skewed distribution and therefore log-transform was used for normalization purposes for these two variables. Feature scaling was also used for all continuous variables.

We trained a logistic regression (LR), a random forest (RF) and an eXtreme Gradient Boosting (XGB) model on the training set on a 10-fold cross validation scheme for parameter optimization. The optimized models were then fitted on the whole training set. A mean-averaging ensemble model was then built on the baseline models' predictions for improved generalizability, robustness and reduced biased predictions. The prediction output of each model regards scaled probabilities that fall within the range of 0–1. Its classification performance was evaluated on the held-out test set. Number of overtreatments and undertreatments by following the model's output, with ROR/Prosigna recommendation as ground truth, are reported for the ensemble model. The model's classification performance is expressed as area under the receiver operating characteristics curve (AUC), with corresponding confidence intervals (CI) using DeLong's method. In addition, we estimated optimal cut-offs for low and high-risk stratification based on the model's predictions in the training set and validated in the testing set. We compared the number of undertreatments, overtreatments and intermediate risk patients that would need to be tested with GEP when using the model or the clinical surrogate definitions of subtypes that are used in the current Swedish guidelines. The classification performance and treatment recommendation analysis of all individual models are presented in Supplementary Figs. 1–3 for the reader to compare with the presented ensemble.

### 2.3 Clinical risk definitions

The performance of the model in relation to clinical practice according to current guidelines was compared also to three clinical risk assessment tools, again using ROR as “ground truth” in terms of risk. The clinical surrogate definitions for breast cancer subtype (Luminal A-like and Luminal B-like) were based on grade, Ki67 using the International Ki67 in Breast Cancer Working Group recommendations [7], and progesterone receptor expression, and generally follow the St Gallen surrogate definitions of breast cancer subtypes (Supplementary Table 3) [17].

Clinical Treatment Score 5 (CTS5) is an online tool that predicts risk for delayed distant breast cancer recurrence for patients that are disease free after 5 years of endocrine therapy. Patients are classified as having low, intermediate, or high risk for distant recurrence by using recurrence thresholds at 5 % and 10 %, using age, nodal status, grade, and tumor size as variables [18].

In the MINDACT [5] and TAILORx trials [19], the clinical high risk definition for node-negative breast cancer was based on tumor size and grade: high risk was defined as grade 3 tumors larger than 1 cm, or grade 2 and larger than 2 cm, or grade 1 and larger than 3 cm. Low risk according to this definition corresponds to 10-year breast cancer–specific survival of at least 92 % for patients with HR + disease treated with postoperative endocrine therapy alone.

The Nottingham Prognostic Index (NPI) is a well-validated prognostic formula that consists of tumor size, nodal stage and tumor grade [20]. Its use for patient selection for testing with GEP using a cutoff of >3.4 for node negative breast cancer is supported by the United Kingdom National Institute for Health and Care guidelines [21].

The Plan B trial evaluated the clinical utility of Oncotype Dx for chemotherapy de-escalation for patients with luminal disease and no nodal metastasis or low nodal burden. In a correlative analysis, Oncotype Dx provided most prognostic information for patients with Ki67 between 10 % and 40 % [22].

### 2.4 Statistical analysis

Categorical variables were summarized using proportions and compared with Pearson's chi-squared test, while continuous variables were summarized using medians and compared with the Mann-Whitney test. The concordance between risk stratification schemes and ROR/Prosigna was reported by the number of over- and undertreatments for each scheme compared to the ROR/Prosigna result, and with Cohen's kappa. Within the scope of this study, the indication for chemotherapy according to ROR/Prosigna concerned patients with intermediate and high ROR (ROR >40) [23]. Recognizing the uncertainty on the optimal management of ROR intermediate patients, we retrained the model and performed additional sensitivity analysis with only high ROR patients (ROR >60) receiving an indication for chemotherapy, while intermediate/low ROR patients (ROR ≤60) did not. Statistical analysis was performed in SPSS version 25.0 (IBM, Armonk, NY, USA).

## 3 Results

### 3.1 Development and validation of a machine-learning model for prediction of ROR/Prosigna recommendation

The characteristics of the 348 postmenopausal women with primary resected pT1c-T3, HR+/HER2-and node negative breast cancer tested for ROR/Prosigna and included in the model are described in Table 1.

**Table 1:** Clinical characteristics of patients included in the study cohort with available ROR/Prosigna results.

	Number of patients (%)
**Number of patients**	348 (100)
**Age, median (range)**	65 (41–84)
**Tumor size in mm, median (range)**	18 (11–95)
**Tumor grade**	
Grade I	13 (3.7)
Grade II	281 (80.7)
Grade III	54 (15.5)
**Ki67 %, median (range)**	25 (2–85)
**Progesterone receptor expression**	
≥20 %	265 (76.1)
<20 %	83 (23.8)
**Clinical subtype**^a^	
Luminal A-like	184 (52.8)
Luminal B-like	164 (47.1)
**Molecular subtype**^b^	
Luminal A	203 (58.3)
Luminal B	139 (39.9)
HER2-enriched	5 (1.4)
Unknown	1 (0.2)
**Risk of Recurrence group**^b^	
Low risk (ROR 0–40)	141 (40.5)
Intermediate risk (ROR 41–60)	142 (40.8)
High risk (ROR 61–100)	65 (18.6)

a Defined as in Supplementary Table 3.

b According to Prosigna assay.

**Fig. 2:**
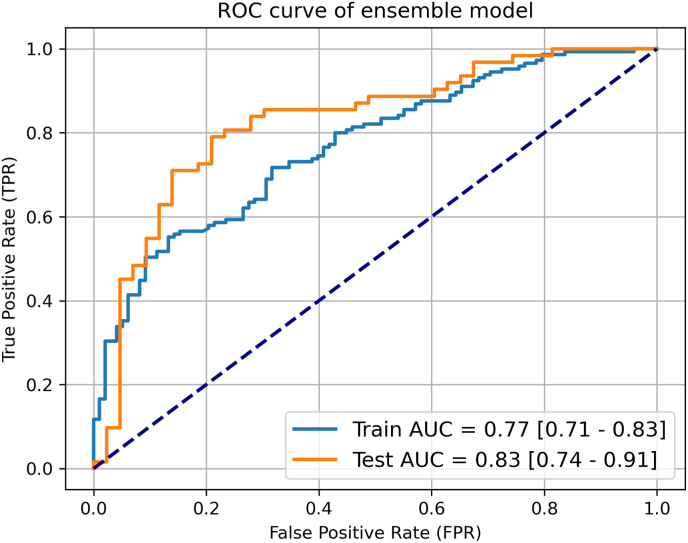
Area under the receiver operating characteristic curves of the averaging ensemble model for the prediction of ROR/Prosigna outcome in the training (blue) and the validation cohort (orange). The dark dotted line represents a random classifier for reference. (For interpretation of the references to colour in this figure legend, the reader is referred to the Web version of this article.)

The training cohort comprised 243 patients, of which 98 had low ROR and 145 intermediate/high ROR. The respective number of patients for the validation cohort were 105 in total, 43 low and 62 intermediate/high ROR. The performance of the final ensemble model in the training and the validation cohorts is shown in Fig. 2, achieving an AUC of 0.77 (95 % CI 0.71–0.83) in the training and 0.83 (95 % CI 0.74–0.91) in the held-out validation cohort for predicting the ROR/Prosigna recommendation. The classification performance of the individual ensemble model components is presented in Supplementary Fig. 4, with LR and RF showing similar results and improved performance on the test set compared to the training set, while XGB demonstrates consistent performance across both sets. This highlights the ensemble model as a more robust, accurate, and reliable option, outperforming the individual models by leveraging their combined strengths and providing better generalization.

**Fig. 3:**
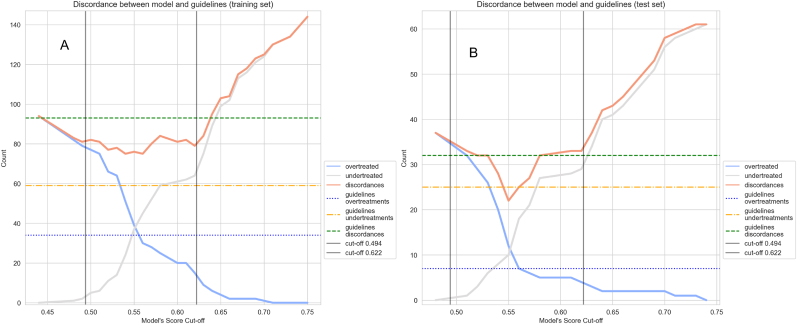
Plots depicting the number of undertreatments, overtreatments and overall discordances compared with current guidelines and using ROR/Prosigna outcome as ground truth, across model cut-off values in the training (A) and the validation cohort (B). The vertical lines represent the model-based risk stratification cut-off values, optimized on the training set, for reference.

To better illustrate the performance of the model, it was compared to the clinical subtype surrogate definitions according to grade, Ki67 and progesterone receptor expression. The overall percent agreement between clinical and molecular subtypes was 65.2 % (Cohen's kappa 0.29). Applying the classification to Luminal A-like and Luminal B-like breast cancer for decision to administer chemotherapy or not would lead, compared to testing every patient with ROR/Prosigna, to the same management in 64.1 % of all patients (n = 223), to undertreatment to 24.1 % (n = 84) and overtreatment to 11.8 % (n = 41). In comparison, the model could further decrease the number of discordant cases with ROR/Prosigna by 27, leading to an overall percent agreement with ROR/Prosigna of 71.8 % (Cohen's kappa 0.41). Model performance across various thresholds is shown in Fig. 3.

### 3.2 Clinical impact of the machine-learning model

We then explored the potential clinical impact of the model when setting cut-offs to distinguish low, intermediate, and high-risk groups. The output here is instruction to the treating physician to proceed with chemotherapy without testing (high risk), test with GEP (intermediate risk) or omit chemotherapy (low risk). This output was compared to the current management of initial risk stratification using anatomic stage and the surrogate subtype definitions. The performance of the latter is shown in Table 2, with over- and undertreatments at 6 % (n = 21) and 0.8 % (n = 3) respectively with ROR/Prosigna outcome as ground truth, while 60.9 % of patients (n = 212) were classified as intermediate risk that need to be tested with GEP (Cohen's kappa 0.41).

**Table 2:** Performance of five risk classifications compared with testing all patients with ROR/Prosigna. Correct classification includes no chemotherapy indication for ROR low patients and chemotherapy indication for ROR intermediate/high patients. Model performance is demonstrated separately for the training (n = 243) and validation (n = 105) cohorts, whereas the performance of the other classifications is demonstrated for the pooled cohort (n = 348).

	Clinical guidelines 2022	CTS5	Ki67 10 %/40 % cut-offs	MINDACT/TailorX groups	Nottingham Prognostic Index	Model training	Model validation
**Correct clinical classification**	112 (32.1 %)	83 (23.8 %)	44 (12.6 %)	194 (55.7 %)	156 (44.8 %)	101 (41.5 %)	43 (40.9 %)
**Overtreated**	21 (6.0 %)	9 (2.5 %)	2 (0.5 %)	56 (16.1 %)	1 (0.3 %)	14 (5.7 %)	4 (3.8 %)
**Undertreated**	3 (0.8 %)	76 (21.8 %)	1 (0.3 %)	98 (28.1 %)	191 (54.9 %)	2 (0.8 %)	1 (0.9 %)
**Cohen's κ**	0.41	0.09	0.83	0.12	0.06	0.61	0.73
**Intermediate risk - for testing with Prosigna**	212 (60.9 %)	180 (51.7 %)	301 (86.5 %)	NA	NA	126 (51.8 %)	57 (54.2 %)

Lower and upper cut-offs of 0.494 and 0.622 were derived in the training cohort for the model to aim for similar proportions of undertreated and overtreated patients as with the clinical practice guidelines and were then validated in the testing cohort. Compared with current guidelines, the model improved both correct classification (Cohen's kappa 0.61 in the training and 0.73 in the validation cohort versus 0.41 for the guidelines) and decreased the proportion of patients classified as intermediate risk with an indication for GEP (51.8 % in the training and 54.2 % in the validation cohort versus 60.9 % for the guidelines). In other words, use of the model led to relative reductions of the number of ROR/Prosigna tests by 13.7 % and the number of overtreated patients by 14.3 % compared to current practice, without increasing undertreatments.

Similar results were observed for all individual models, except for XGB, which exhibited the greatest variability by predicting a significantly higher number of patients as intermediate risk compared to the other models (Supplementary Figs. 1–3 and Supplementary Table 4). Overall, the ensemble model demonstrated strong and accurate performance, effectively balancing the strengths of the individual models.

### 3.3 Risk stratification schemes to improve patient selection for ROR/Prosigna testing and comparison with the machine learning model

We then evaluated the performance of five different risk classifications: the current guidelines in use which are based on the clinical subtype surrogate definitions according to grade and Ki67, and recommend GEP for patients in the intermediate risk group (Supplementary Table 2); predefined Ki67 cut-offs at 10 % and 40 % according to a previous study from the Plan B trial [22]; the CTS5 online tool; the risk definitions from the MINDACT and TailorX trials; and NPI. In summary, risk classifications varied in terms of performance, either leading to excessive undertreatments by recommending no chemotherapy and no testing in patients that were ROR intermediate/high, as with CTS5 and NPI, or in the case of the Plan B Ki67 cut-offs to large intermediate group (86 % of the entire cohort) that would need to be tested, despite limiting over- and undertreatments. As such, the proposed machine learning model led to the best combination of number of patients that would be undertreated, overtreated and would need to be tested, using ROR/Prosigna outcome as the ground truth. These results are shown in Table 2.

### 3.4 Sensitivity analysis

Sensitivity analysis with a ground truth of only ROR high patients receiving a chemotherapy indication was performed for all risk stratifications, including the ensemble machine learning model using the same procedures. Model cut-offs of 0.0668 and 0.1596 were derived in the training cohort that resulted in similar under- and overtreatments as according to clinical practice guidelines and were then validated in the testing cohort. The results remained unchanged, with substantial improvements in terms of improved correct classification (Cohen's kappa 0.26 in the training and 0.32 in the validation cohort versus 0.06 for the clinical guidelines) and decrease in the percentage of intermediate risk patients needing GEP (44.0 % in the training and 37.1 % in the validation cohort versus 60.9 % for the clinical guidelines) (Supplementary Table 5). Compared to current practice, the model led to relative reductions of number of patients needed to test with ROR/Prosigna by 31.2 % and overtreated patients by 2.5 % without increasing undertreatments.

## 4 Discussion

Limiting GEP only to breast cancer patients with intermediate clinical risk, a vaguely defined population, can lead to unnecessary over- and undertreatment. In contrast, wide testing with no patient selection can lead to increased costs. As the molecular drivers of available GEP differ [24], previous results on optimizing RS/Oncotype Dx testing may not be applicable for all available tools. To our knowledge, this is the first attempt to optimize ROR/Prosigna testing by using a machine-learning based model comprising simple, readily available clinical variables that outperformed other risk classifications that employ the same prognostic factors.

Use of commercial gene signatures in early breast cancer has been shown to be cost-effective [[25], [26], [27]]. However, inappropriate study design and assumptions may have affected the studies’ conclusions [25,28]. Logistic regression models were therefore initially developed [10] and further refined [11] to limit testing and health-related costs without jeopardizing patient outcomes. In this study, we developed and validated a machine-learning based model that decreased discordance with ROR/Prosigna outcome. To assess its performance, we evaluated previously validated risk classifications and determined that they were inadequately precise for clinical translation since they either required extensive testing of large intermediate risk populations or led to considerable over- and undertreatment. By setting externally validated cut-offs, the model could improve correct risk classification, while at the same time limiting the proportion of intermediate risk patients. These results demonstrate the feasibility of easy-to-implement machine-learning models that leverage readily available prognostic factors as a tool to decrease unnecessary healthcare costs through more precise selection of candidates for GEP.

Study limitations include limited size of the study cohort comprising preselected, largely intermediate risk patients which limits the generalizability of our results, even though the represented population is approximately one third of the entire Swedish population. In addition, the participating centers follow relatively homogeneous clinical routine and the same national guidelines, which underscores the need to validate the model in non-Swedish cohorts. Furthermore, there is no clear guidance on the approach to patients with intermediate ROR. In this study we assumed they would be treated with chemotherapy since avoidance of undertreatment was valued higher than risk for overtreatment. However, we also performed sensitivity analysis with high ROR being the indication to treat and our results and conclusions remained unchanged. Finally, median follow-up of these patients is short since ROR/Prosigna has only been recommended for use in Sweden since 2020. As a result, the prognostic performance of the model is yet unclear and will be the focus of a future study as follow-up of these patients continues.

In conclusion, existing clinical risk definitions were imprecise in identifying postmenopausal patients with HR+/HER2-and node-negative breast cancer not needing ROR/Prosigna testing. In contrast, a simple machine-learning based model outperformed these risk definitions and, pending further validation, could lead to improved patient selection, thus limiting both testing- and overtreatment-associated costs.

## CRediT authorship contribution statement

**Una Kjällquist:** Writing – review & editing, Writing – original draft, Investigation, Formal analysis, Data curation, Conceptualization. **Nikos Tsiknakis:** Writing – review & editing, Writing – original draft, Visualization, Validation, Software, Investigation, Formal analysis, Data curation. **Balazs Acs:** Writing – review & editing, Investigation. **Sara Margolin:** Writing – review & editing, Investigation. **Luisa Edman Kessler:** Writing – review & editing, Investigation, Conceptualization. **Scarlett Levy:** Writing – review & editing, Investigation. **Maria Ekholm:** Writing – review & editing, Investigation. **Christine Lundgren:** Writing – review & editing, Investigation. **Erik Olsson:** Writing – review & editing, Investigation. **Henrik Lindman:** Writing – review & editing, Investigation. **Antonios Valachis:** Writing – review & editing, Investigation. **Johan Hartman:** Writing – review & editing, Investigation. **Theodoros Foukakis:** Writing – review & editing, Investigation. **Alexios Matikas:** Writing – review & editing, Writing – original draft, Supervision, Investigation, Formal analysis, Conceptualization.

## Data availability

The dataset generated and analyzed during the current study is available from the corresponding authors on reasonable request. The code and trained model described in the manuscript are available from https://www.github.com/foukakis-grp/ml-opt-guidelines-recurrence-risk, strictly for research use and should not be used for clinical decision making.

## Ethics approval

The study was approved by Swedish ethical review authority (application registry numbers Dnr 2019-01908, Dnr 2020–03903 and Dnr 2023-07300-02). The study is a retrospective non-interventional study using data from patient records. As such, informed consent was waived by Swedish ethical review authority. A**ll methods were performed in accordance with the relevant guidelines and regulations.**

## Funding

The study did not receive any funding

## Declaration of competing interest

Maria Ekholm: speaker honoraria from AstraZeneca and participation on advisory boards organized by Pfizer, Lilly, and Novarits, all payments made to institution. Henrik Lindman: research funding from 10.13039/100004337Roche and personal fees from Lilly, Daiichi, and 10.13039/100004336Novartis outside the submitted work. Antonis Valachis unrestricted research funding paid to instritution by 10.13039/100004337Roche and 10.13039/100030732MSD. Johan Hartman: research funding to institution from 10.13039/100004336Novartis and Cepheid; leadership and stock ownership at Stratipath AB; honoraria from 10.13039/100004336Novartis, 10.13039/100004334Merck, Lilly and 10.13039/100004319Pfizer. Theodoros Foukakis: institutional fees for consultancy to 10.13039/100004325AstraZeneca, 10.13039/100005564Gilead and Roche; personal fees for consultancy to Affibody, 10.13039/100004319Pfizer, 10.13039/100004336Novartis, 10.13039/100018771Veracyte, Exact Sciences; honoraria from UpToDate; research funding to institution from 10.13039/100004319Pfizer, 10.13039/100004325AstraZeneca, 10.13039/100004336Novartis and 10.13039/100018771Veracyte. Alexios Matikas: speaker/consultancy (no personal fees) to 10.13039/100018771Veracyte, 10.13039/100004337Roche, Seagen; research funding paid to institution by 10.13039/100004334Merck, 10.13039/100004325AstraZeneca, 10.13039/100004336Novartis, 10.13039/100018771Veracyte. All the other authors had no potential conflicts of interest to disclose.
