# Supplementary material for: Optimization of guidelines for Risk Of Recurrence/Prosigna testing using a machine learning model: a Swedish multicenter study
Source: Breast. 2025 May 7;82:104489. doi: 10.1016/j.breast.2025.104489 (PMC12139505; doi:10.1016/j.breast.2025.104489)
Supplement: Multimedia component 1 [file mmc1.docx]

**Optimization of guidelines for Risk of Recurrence/Prosigna testing using a machine learning model: a Swedish multicenter study**

Una Kjällquist ^1,2#^, Nikos Tsiknakis ^1#^, Balazs Acs ^1,2^, Sara Margolin ^3,4^, Luisa Edman Kessler ^5^, Scarlett Levy ^5^, Maria Ekholm ^6,7^, Christine Lundgren ^6,7^, Erik Olsson ^8^, Henrik Lindman ^8^, Antonios Valachis ^9^, Johan Hartman ^1,3^, Theodoros Foukakis ^1,2^, Alexios Matikas ^1,2^

^#^ The two first authors contributed equally

**Supplementary Tables 1 – 5**

* Pathology lab specific Ki67 cut-of for low, intermediate and high expression. Karolinska, Södersjukhuset and St:Göran hospital low 0–14%, intermediate 15-22%, high 23-100%; Jönköping hospital low 0–11%, intermediate 12-21%, high 22-100%;

Akademiska hospital low 0–14%, intermediate 15-23%, high 24-100%.

**Supplementary Table S4**. Treatment recommendation per model

| **Supplementary Table 5.** Sensitivity analysis based on chemotherapy indication for ROR high patients only. Performance of five risk classifications compared with testing all patients with ROR/Prosigna. Correct classification includes no chemotherapy indication for ROR low/intermediate patients and chemotherapy indication for ROR high patients. Model performance is demonstrated separately for the training (n=243) and validation (n=105) cohorts, whereas the performance of the other classifications is demonstrated for the pooled cohort (n=348) | | | | | | | |
| --- | --- | --- | --- | --- | --- | --- | --- |
|  | **Clinical guidelines 2022** | **CTS5** | **Ki67 10%/40% cut-offs** | **MINDACT/ TailorX groups** | **Nottingham Prognostic Index** | **Model training** | **Model validation** |
| **Correct clinical classification** | 53 (15.2%) | 129 (37.0%) | 25 (7.1%) | 214 (61.4%) | 288 (82.7%) | 78 (32.1%) | 43 (40.9%) |
| **Overtreated** | 81 (23.2%) | 19 (5.4%) | 21 (6.0%) | 117 (33.6%) | 6 (2.1%) | 57 (23.4%) | 22 (20.9%) |
| **Undertreated** | 2 (0.5%) | 20 (5.7%) | 1 (0.2%) | 17 (4.8%) | 54 (13.9%) | 1 (0.4%) | 1 (0.9%) |
| **Cohen’s κ** | 0.06 | 0.25 | 0.20 | 0.20 | 0.21 | 0.26 | 0.32 |
| **Intermediate risk - for testing with Prosigna** | 212 (60.9%) | 180 (51.7%) | 301 (86.4%) | NA | NA | 107 (44.0%) | 39 (37.1%) |

**Supplementary Figures 1 – 4**

**Supplementary Figure S1**

Plots for the **Logistic Regression** model depicting the number of undertreatments, overtreatments and overall discordances compared with current guidelines and using ROR/Prosigna outcome as ground truth, across model cut-off values in the training (A) and the validation cohort (B)

**Supplementary Figure S2**

Plots for the **Random Forest** model depicting the number of undertreatments, overtreatments and overall discordances compared with current guidelines and using ROR/Prosigna outcome as ground truth, across model cut-off values in the training (A) and the validation cohort (B)

**Supplementary Figure S3**

Plots for the **eXtreme Gradient Boost** model depicting the number of undertreatments, overtreatments and overall discordances compared with current guidelines and using ROR/Prosigna outcome as ground truth, across model cut-off values in the training (A) and the validation cohort (B)

**Supplementary Figure S4**

Area under the receiver operating characteristic curves for the prediction of ROR/Prosigna outcome in the training (A) and the validation cohort (B). LR: logistic regression (blue); RF: random forest (orange); XGB: extreme gradient boosting (green); The dark dotted line represents a random classifier for reference.
